# Immunonutrition for the Management of Postsurgery GI Cancer Patients

**DOI:** 10.3390/nu18081229

**Published:** 2026-04-14

**Authors:** Annalisa Pezzoli, Marialaura Scarcella, Giacomo Recanatini, Arianna Di Bernardino, Carlo Rasetti, Domenico Morano, Jan Tack, Ludovico Abenavoli, Emidio Scarpellini

**Affiliations:** 1 Internal and Nutritional Unit, “Madonna del Soccorso” General Hospital, 63074 San Benedetto del Tronto, Italy; pezzoliannalisa@gmail.com (A.P.); giacomo.recanatini@sanita.marche.it (G.R.); arianna.diberadino@sanita.marche.it (A.D.B.); carlo.rasetti1@sanita.marche.it (C.R.); 2 Anesthesia, Intensive Care and Nutritional Science, “Santa Maria” Hospital, Terni and Nutritional Residency Program, Perugia University, 06121 Perugia, Italy; m.scarcella@aospterni.it; 3 Department of Health Sciences, University “Magna Graecia”, 88100 Catanzaro, Italy; domenico.morano@unicz.it (D.M.); l.abenavoli@unicz.it (L.A.); 4 Center for Chronic Liver Diseases, “Renato Dulbecco” University Hospital, 88100 Catanzaro, Italy; 5 Translational Research in Gastrointestinal Disorders (T.A.R.G.I.D.), Gasthuisberg University Hospital, KU Leuven, 3000 Leuven, Belgium; jan.tack@med.kuleuven.be

**Keywords:** enteral immunonutrition, gastrointestinal cancer, immune response, inflammation, postoperative complication

## Abstract

Postoperative complications in gastrointestinal (GI) cancer patients remain a significant challenge for physicians. It leads to increased morbidity, prolonged hospital stays, and higher healthcare costs. Enteral immunonutrition (EIN) has emerged as a promising add-on treatment to modulate immune response following surgery. In fact, it reduces inflammation and promotes patients’ recovery. Indeed, the literature data on its real clinical impact for the patients are inconsistent and, yet, poorly investigated. Thus, the aim of this review was to narratively assess the current evidence for the use of EIN in postoperative GI cancer patients, evaluating the effect on clinical and immunological outcomes of patients. Therefore, a literature search was conducted using the following keywords and associations: enteral immunonutrition, gastrointestinal cancer, immune response, inflammation, and postoperative complication. GI cancers, mainly esophageal and gastric cancer, represent a significant global health burden, characterized by high incidence and mortality rates. The complex interplay between tumor progression, systemic inflammation, and host nutritional status profoundly impacts patient outcomes. Traditional cancer treatments are effective and often lead to severe side effects. The latter includes malnutrition and immunosuppression and can significantly affect patients’ recovery. In recent times, the concept of immunonutrition has emerged as a promising add-on therapy able to consensually modulate immune response and improve nutritional status. Several studies and meta-analyses suggest that EIN can reduce postoperative infections (e.g., wound infections and sepsis incidence), shorten hospital stays, and improve overall outcomes in GI cancer surgery patients vs. standard enteral feeding. EIN is a promising add-on approach for the management of postoperative GI cancer patients. It can significantly reduce postoperative complications and enhance their recovery. However, the result seems consistent for gastric but not yet esophageal cancer patients. EIN shows high tolerance and a high safety profile.

## 1. Introduction

Surgical treatment for gastrointestinal (GI) cancers is often associated with significant postoperative complications, including localized/systemic infections, poor wound healing and longer hospital stay. One of the key factors influencing these outcomes is the patients’ nutritional and immunological status before and after surgery [1]. In recent years, enteral immunonutrition (EIN) has gained increasing attention from scientists and clinicians. It is a specific nutrition delivered through the gut and enriched with nutrients that actively modulate the immune system (e.g., arginine, omega-3 fatty acids, glutamine, and nucleotides) [2]. Arginine can help wound healing through the up-regulation of T-lymphocytes that “sense” and counteract the infections. Moreover, they help in the recognition of cancer cells [3]. Omega-3 fatty acids from fish oil can stabilize and down-regulate the cytokine production, making the immune response more efficient and less detrimental for human tissues [4]. Glutamine helps maintain the integrity of the intestinal barrier and supports the metabolism of immune cells [5]. Finally, bioactive nucleotides contribute to tissue cell repair and proliferation [6].

In the perioperative surgery period (including both pre- and postoperative ones), EIN administration has been shown to be associated with a reduced number and severity of postoperative complications [7,8]. In fact, several studies and meta-analyses have shown that EIN can significantly lower the risk of infections, especially from the surgical site, and shorten the length of hospital stay. In addition, EIN use has also been associated with fewer anastomotic leaks in both upper and lower GI cancer patients [9]. Interestingly, EIN can help prevent the pathophysiologic “immunosuppression” following surgery [9]. However, we must recognize that these studies are varied in terms of experimental setup and the population under study. It is not yet clear which patients can benefit the most from EIN, what the optimal immunonutrient combination is, and whether EIN can fit within the wider Enhanced Recovery After Surgery (ERAS) scheme.

Thus, we performed a narrative review of literature on the concept of immunonutrition (precisely, EIN), its perioperative use in upper and lower GI cancer patients undergoing surgery and its impact on postsurgical complications.

## 2. Materials and Methods

We conducted a search on PubMed and Medline for literature data (namely original articles, reviews, meta-analyses, and case series) using the following keywords, their acronyms, and their associations (e.g., “and”): enteral immunonutrition, gastrointestinal cancer, immune response, inflammation, and postoperative complication. In addition, preliminary evidence from abstracts from the main national and international gastroenterological and nutrition meetings (e.g., United European Gastroenterology Week, Digestive Disease Week, Italian Society of Metabolism and Artificial Nutrition (SINPE) national meeting, and European Society of Metabolism and Artificial Nutrition (ESPEN) international congress) was also included. The articles found in the search were reviewed by two of the authors (E.S. and A.P.). The last Medline search was conducted on 31 December 2025.

In detail, a comprehensive search strategy was constructed using MEDLINE, Embase, and Web of Science from January 2000 to December 2025. Search terms included combinations of “enteral immunonutrition”, “gastrointestinal cancer”, “immune response”, “inflammation”, and “postoperative complication”.

The search incorporated both MeSH terms and free-text keywords to maximize retrieval sensitivity.

Reference lists of key articles were screened to identify additional relevant publications. Studies were included if they involved adult GI cancer patients and examined at least one of the following: clinical outcome, mortality, infectious and non-infectious surgical complications, hospital length of stay, side effects/adverse events upon EIN perioperative administration.

We excluded pediatric studies, case reports, non-surgical populations, and articles lacking nutritional and clinical relevance. Studies not written in the English language were also excluded.

Extracted variables included study design, patient population, cancer type and localization, type of surgery, type, dosage, and duration of EIN formula (where available) administration, infectious and non-infectious surgical complications, mortality, length of hospital stay, side-effects/adverse events upon EIN administration.

Due to heterogeneity of study protocols, patient population, type, dosage and duration of EIN formula administration, cancer type and localization, surgical procedures, and surgical outcome definitions, quantitative pooling was not feasible. Instead, we performed a qualitative synthesis centered on identifying recurring surgical patients’ outcomes and integrating them into a cohesive conceptual framework. In parallel, we examined barriers to implementation and explored potential avenues for future research required to validate an EIN-driven nutritional precision protocol in GI cancer patients undergoing surgery.

**PICO Framework (Population, Intervention, Comparator, and Outcomes)**

PICO Framework

To structure the research question guiding this narrative review, a PICO framework was applied to ensure clarity, transparency, and methodological rigor while maintaining the flexibility appropriate for a narrative synthesis [10].

P—Population

Adult GI cancer patients undergoing surgery are administered the EIN formula in the perioperative period. Studies were eligible if they involved patients undergoing mortality rate, infectious and non-infectious surgical complications follow-up, and hospital length of stay assessment.

I—Intervention/Exposure

Any of the following interventions or monitoring modalities are applicable:

Perioperative EIN administration to GI cancer patients undergoing surgery.

C—Comparator

Comparators varied depending on study design and included the following:

- Traditional predictive equations (e.g., Harris–Benedict, Penn State, and Ireton-Jones).
- standard enteral nutrition formula.

Given the heterogeneity inherent to study protocols, patient population, type, dosage and duration of EIN formula administration, cancer type and localization, surgical procedures and surgical outcome definitions, narrative comparison across surgical patients’ outcomes, rather than direct head-to-head trials, was prioritized.

O—Outcomes

Eligible outcomes included clinical and nutritional endpoints:

Clinical outcomes:

- Infectious and non-infectious surgical complications [11];
- Mortality (surgery ward, or follow-up);
- Length of hospital stay;
- Incidence of side effects/adverse events upon EIN administration.

Nutritional outcomes:

- Caloric adequacy and length/timing of EIN administration;
- Protein adequacy;
- EIN Feeding tolerance;
- Changes in lean mass or functional muscle indices.

Purpose of PICO in a Narrative Review

While narrative reviews do not apply the PICO model as strictly as systematic reviews, incorporating a PICO framework provides the following [12]:

- Clear definition of the investigative scope;
- Transparent inclusion logic;
- Consistent handling of heterogeneous evidence;
- Improved reproducibility and methodological credibility.

Formal risk of bias (RoB) assessment evaluated the methodological quality of studies to determine if their results can be trusted. It involved assessing specific domains (randomization, blinding, and attrition) using standardized tools (namely, RoB 2) to categorize studies as having low, high, or unclear risk.

Quality assessment of meta-analysis involved evaluating both the methodological rigor of the review process itself and the risk of bias in the included studies. Key steps included assessing internal validity (bias risk), ensuring transparent methodology, and checking for publication bias.

This review was conducted using a structured, transparent methodology inspired by PRISMA principles, while maintaining the flexibility appropriate to a narrative review.

The goal was to ensure comprehensive coverage of the literature related to EIN perioperative administration to GI cancer patients undergoing surgery, without the restrictions imposed by a strictly systematic design [13].

Search Strategy

A comprehensive literature search was performed across MEDLINE (via PubMed), Embase, and Web of Science, covering the period from January 2000 to December 2025. The search combined controlled vocabulary terms (MeSH and Emtree) with free-text keywords to maximize sensitivity. Search terms included the following:

- “enteral immunonutrition” OR “immunonutrition”;
- “GI cancer” OR “ upper and lower GI cancer”;
- “immune response” OR “ immunity”;
- “inflammation” OR “inflammatory response”;
- “surgery outcome” OR “clinical outcome”.

Boolean operators and proximity filters were applied to refine results. No language restrictions were imposed initially; however, only English-language publications were retained during screening.

Eligibility Criteria

Studies were considered eligible if they met the following criteria:

1. Population: Adult GI cancer patients undergoing surgery.
2. Intervention/Exposure: Perioperative EIN.
3. Outcomes: Nutritional status, mortality, infectious and non-infectious surgical complications, or hospital length of stay.
4. Study Design: Randomized trials, observational studies, cohort analyses, cross-sectional studies, validation studies, case series.

The following were excluded:

- Pediatric studies;
- Case reports or case series with n < 10;
- Non-human studies;
- Articles without relevance to nutritional clinical outcome monitoring;
- Purely mechanistic papers with no clinical application.

Study Selection

Titles and abstracts (including congress abstracts) were first screened for relevance by two independent reviewers (E.S. and A.P.). Full texts were subsequently assessed for compliance with eligibility criteria. Disagreements were resolved through discussion and consensus. Although a formal PRISMA flow diagram was not constructed, the selection process followed a structured approach similar to systematic reviews.

Data Extraction and Synthesis

Data extracted from the included studies encompassed the following:

- Study design and sample characteristics;
- Type and localization of cancer;
- Type of surgery;
- Type, dosage and duration of EIN formula (where available) administration;
- Mortality, infectious and non-infectious surgical complications, length of hospital stay;
- Side-effects/adverse events.

**Figure 1:**
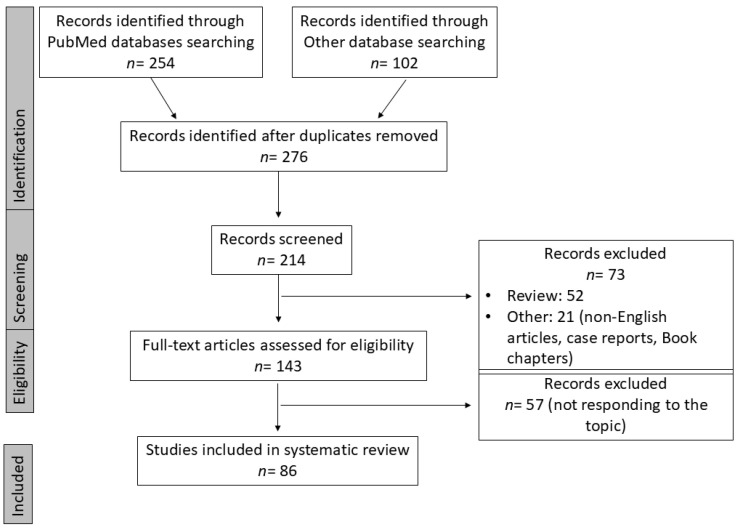
Flow-chart of review process of articles inspired by PRISMA guidelines.

Because of heterogeneity in study protocols, patient population, type, dosage and duration of EIN formula administration, cancer type and localization, surgical procedures and surgical outcome definitions, quantitative synthesis was not feasible. Instead, a qualitative, narrative synthesis approach was adopted, prioritizing recurring surgical patients’ outcomes, converging trends, and future precision nutritional protocol implications (Figure 1).

Risk of Bias and Quality Considerations

Although formal risk-of-bias tools typically used in systematic reviews were not applied, studies were qualitatively evaluated for the following:

- Methodological clarity;
- Appropriateness of measurement protocols;
- Completeness of reporting.

## 3. Results

### 3.1. Immunonutrition Compounds and Immune Effects

#### 3.1.1. Immunonutrition Definition and Immunonutrients

“Immunonutrients” are those nutrients with modulating pharmacologic effects on the immune and inflammatory cascade. More correctly, they are administered in higher-than-normal amounts to modulate the immune system and metabolic response, especially in surgical or critically ill patients. They are able to increase cellular immune response, promoting wound healing, modulating inflammatory responses, and improving intestinal mucosal barrier function [14]. In detail, they can stimulate immune cell activity, reduce hyper-inflammation, and allow operated, damaged tissues to re-establish. Altogether, these can result in enhanced wound healing (e.g., patients undergoing surgery and active cancer treatment (adjuvant/neoadjuvant chemotherapy, immunotherapy, and radiotherapy)) [12]. In fact, the massive and disordered inflammatory response following a stressor like surgery, trauma or infection, can lead to hypermetabolism and depletion of nutrient stores from the body’s tissues. Moreover, the high inflammatory levels due to a pro-inflammatory cytokine storm can, paradoxically, lead to an immunosuppressive state that favors cancer cell proliferation. In fact, malnourished patients have reduced immune system functioning because of acute phase protein depletion [12,15].

Several nutrients can modulate immune response, rebalancing the inflammatory response. Zinc, epigallocatechin gallate (EGCG), omega-3 polyunsaturated fatty acids, and probiotics have immunomodulant properties [16,17].

In GI tract cancer surgery patients, surgical complications mainly include opportunistic infections and poor wound healing. Their rate is higher when the immune system is dysfunctional [18]. Dietary modulation of the immune system has been recognized as a promising add-on treatment for surgically treated cancer patients, leading to the development of newer food products [19]. Although most of the currently available literature on immunonutrition use focuses on treated cancer patients, the greatest potential of these nutrients can be found for cancer prevention [20]. Immunonutrients can be administered alone or through several available combinations.

##### Key Immunonutrition Compounds Include the Following

- *Arginine:* an amino acid crucial for T-cell function and wound healing. In detail, it promotes T-cell proliferation and macrophage activity. It serves as a precursor to nitric oxide (NO) synthesis, responsible for vasodilation, tissue leukocyte infiltration and microbial killing [21]. Arginine supports collagen synthesis via conversion to proline and hydroxyproline for wound-repairing processes [18].

- *Omega-3 polyunsaturated fatty acids*: in general, PUFA sources include fatty fish (salmon, mackerel, and sardines), seeds (flaxseed and chia), walnuts, and vegetable oils (soybean, sunflower, and corn). These provide essential omega-3 and omega-6 fats necessary for neuronal and visual function. Flaxseed oil offers high alpha-linolenic fatty acid (ALA), while fish and algae are excellent sources of eicosapentaenoic acid and docosahexaenoic acid (EPA and DHA, respectively) [19]. In detail, certain vegetable-derived oils (e.g., safflower, soy, corn, and sunflower) contain high levels of the omega-6 polyunsaturated fatty acid (*n*-6 PUFA) and linoleic acid [19]. On the other hand, linseed, rapeseed and walnut oils contain high levels of linolenic acid, belonging to the *n*-3 PUFA family [19,20]. Both can be metabolized to the long-chain PUFA, arachidonic acid (*n*-6) or EPA (*n*-3). Moreover, lean meat and fish contain high amounts of the long-chain *n*-6 PUFA; fatty fish like salmon, tuna and herring contain the long-chain *n*-3 PUFA [19,20]. It has been observed that populations consuming high levels of oily fish have significantly lower cancer incidence vs. the general population [22]. This preventive cancer development effect has been related to the high *n*-3 to *n*-6 PUFA ratio within their diet [19].

*N*-3 PUFA immunomodulant effect can be explained by their role in the building of inflammatory cell membranes and in the modulation of proinflammatory cytokine production with inhibition of NK-kB signaling. Specifically, they can reduce eicosanoid production (anti-inflammatory action) and actively support macrophages and neutrophil activation in response to pathogenic stimuli [23].

- *Polyphenols and Epigallocatechin- 3-gallate (EGCG):* plant polyphenols are a group of chemicals with a beneficial but non-essential role in human nutrition. Their group has biological effects, including antioxidant, antimutagenic and anti-inflammatory ones [24]. Some polyphenols can reduce the endothelial cell expression of proinflammatory cytokines and adhesion molecules with lesser monocyte infiltration. Interestingly, they can slow and, perhaps, prevent cancer growth through a significant inhibition of neoangiogenesis [25]. EGCG is the major constituent of green tea and has the most significant anti-inflammatory and cancer-preventive capabilities.

- *Glutamine:* it is the most abundant free amino acid in the diet and, above all, becomes essential upon trauma/surgery. Specifically, it is the brick for antibodies’ production, T-B cell maturation, cytokines’ expression (e.g., IL-2, IFN-γ, and TNF-α) [26,27]. In addition, it is the precursor of glutathione synthesis. The latter has marked antioxidant properties and protects tissues from oxidative stress, maintaining the integrity of the intestinal barrier [23].

*- Nucleotides* (namely, RNA/DNA agglomerates) are depleted during cells’ stress-induced death. They are able to enhance lymphocytes’ activation, maturation and proliferation. They favor macrophages’ phagocytosis and, last but not least, genome repairing for the effective immune system surveillance [28].

We must note that antioxidants, as well as pre- and probiotics, cannot be strictly regarded as typical immunonutrients. However, we mentioned them because of the emerging role as add-on components of an immunomodulatory approach for cancer patients under treatment.

*- Antioxidants and Amino Acids*: vitamins C, E, beta carotene, selenium and sulphur contain amino acids supporting a positive redox balance. In detail, they help neutralize reactive oxygen species, control the inflammation, and also upregulate glutathione levels. Thus, they have mainly an anti-inflammatory action. No evidence on the nutritional status of cancer patients has been described [29].

*- Pre- and Probiotics:* beneficial bacteria harboring the GI tract (namely, probiotics) are crucial for its functioning. Probiotics maintain mucosal immunity and help digest carbohydrates normally undigestible within the small bowel. Probiotics can prevent the colonization of the gut by pathogenic microorganisms and sustain the synthesis of the mucus barrier [26,30]. Further, consumption of digestible carbohydrates feeds beneficial bacterial species of gut microbiota (namely, prebiotics) and can fuel a stable immunomodulation within and outside the GI tract [27]. Thus, the use of pre- and probiotics in cancer patients seems to have promising anti-inflammatory and immunomodulating effects.

#### 3.1.2. Immunonutrients and Modulation of Immune/Metabolic Functions: Molecular Pathways

Approximately one-third of cancer-related deaths are due to detrimental lifestyle and, specifically, feeding habits (e.g., low intake of fruits and vegetables, physical inactivity, obesity, and alcohol and tobacco use/abuse) [31]. Thus, nutrition and immune function can be considered closely connected. Moreover, derangements from optimal body weight and dietary deficiency of essential micronutrients can significantly impair immune system response. Interestingly, in cancer patients, the nutritional status can significantly affect patients’ performance status, disease progression and treatment outcomes [32].

The matter of the immune system and cancer natural history is more complex than a dual and bimodal interaction: immune system functioning is responsible for both tumor surveillance and, also promotion [33]. More in detail, effective immune responses mediated by cytotoxic T lymphocytes (CTLs), natural killer (NK) cells, and Th1-response cytokines (e.g., IFN γ) recognize and eliminate early tumor cells. They are cleared through mechanisms like perforin/granzyme release and antigen presentation by dendritic cells. Altogether, these processes resemble “immunosurveillance” [33,34]. They help prevent malignant cells’ transformation and disease progression. Conversely, chronic inflammation, because of prolonged release of pro-inflammatory cytokines (IL 1β, IL 6, and TNF α), reactive oxygen/nitrogen species, and persistent NF κB/STAT signaling, establishes an immunosuppressive tumor microenvironment, TME, that can favor cancer growth and genetic instability [35]. In addition, “metabolic competition” within the TME can further affect immune system activity. In fact, cancer cells deplete amino acids (namely, glutamine, arginine, and tryptophan) and limit the availability for effector T-cells and force immune cells toward exhaustion/down-regulation [33,36].

**Figure 2:**
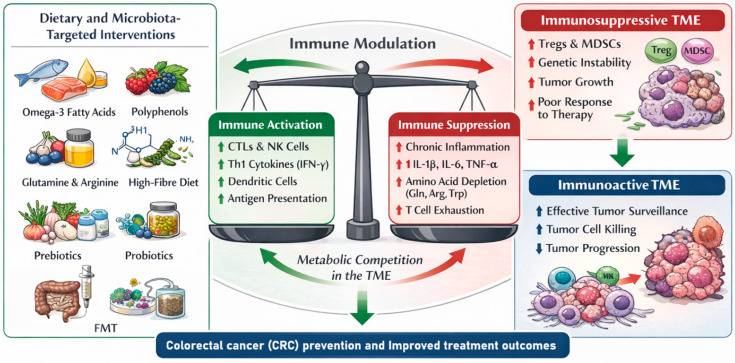
Immunonutrition-driven modulation of immune function in colorectal cancer. Dietary and also the promising microbiota-targeted interventions (e.g., pre- and probiotics, as well as FMT) modulate immune responses and metabolic pathways within the tumor microenvironment (TME). In detail, immunonutrients promote Th1/CTL- and NK-mediated antitumor immunity, reduce chronic inflammation, restore amino acid availability, and counteract immune exhaustion vs. the immunosuppressive environment within the TME. Thus, immunonutrients also generate a “ metabolic competition” for energy sources within the TME. These effects, altogether, contribute to effective tumor “immunosurveillance”, inhibition of cancer progression, and improved response to immunotherapies. Figure legend: CRC, colorectal cancer; TME, tumor microenvironment; CTLs, cytotoxic T lymphocytes; NK cells, natural killer cells; Tregs, regulatory T-cells; MDSCs, Myeloid-Derived Suppressor Cells; IFN-γ, interferon gamma; IL-1β, interleukin-1 beta; IL-6, interleukin-6; TNF-α, tumor necrosis factor alpha; Gln, glutamine; Arg, arginine; Trp, tryptophan; FMT, fecal microbiota transplantation; ↑, upregulation; and ↓, downregulation.

In this frame, dietary and microbial-targeted interventions (e.g., omega-3 fatty acids, polyphenols, glutamine, arginine, and high-fiber diets) can reverse the immunosuppressive mechanisms. In particular, they can reduce inflammation, restore amino acid balance, favor Th1/CTL-mediated responses, and enhance dendritic cells and NK cells activation [34,37]. Interestingly and from a perspective view, modulation of gut microbiota through pre-, pro- and postbiotics, or fecal microbiota transplantation, can significantly improve response to immune checkpoint inhibitors in GI and non-GI cancers through re-establishment of gut eubiosis, enhancing systemic anti-tumor immunity, and reducing Treg/Myeloid-Derived Suppressor Cell (MDSC) ratios [32]. These actions have been extensively described in colorectal cancer (CRC) patients (Figure 2).

#### 3.1.3. Enteral Immunonutrition Mechanisms of Action

Immunonutrients can be administered to the GI tract cancer patients via enteral or parenteral formulas [38].

**Figure 3:**
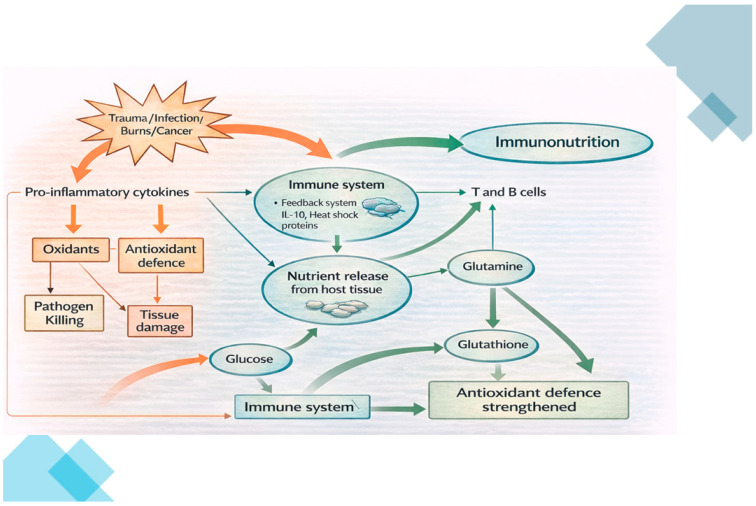
Inflammation-driven metabolic changes can disrupt the body’s antioxidant defenses and affect immune function, potentially compromising host resistance to infections and cancer cells’ surveillance. Immunonutrients can reverse the pro-inflammatory state, maintaining immune system surveillance and tolerance.

Enteral immunonutrition delivers targeted nutrients actively enhancing the immune system functioning (both immuno-surveillance and tolerance) and the nutritional status of the patient within the gut. In particular, enterally administered immunonutrients:

1. *Modulate the inflammatory response:* Omega-3 (and antioxidants) reduce overactive inflammatory responses. The latter decreases tissue damage and accelerates organ healing [33].
2. *Enhance Immune Cells’ Functioning*: Arginine and glutamine act as feeding tools for immune cells like T-cells, B lymphocytes, neutrophils, and macrophages, reinforcing pathogens’ defense and wound repairing [19,20].
3. *Improve microcirculation and allow tissues’ repair*: Nitric oxide derived from arginine enhances tissues’ blood flow. Further, glutamine and nucleotides support DNA/RNA synthesis for tissue regeneration [21,22,23,24].
4. *Maintain Gut Barrier and Redox Homeostasis*: Glutamine (and pre-/probiotics) sustain intestinal lining and tight junctions’ mechanism of action, leading to reduced intestinal permeability to pathogens/procarcinogens. Although not properly defined as immunonutrients, antioxidants counteract oxidative stress and preserve mucosal integrity [24,34] (Figure 3).

#### 3.1.4. The Clinical Application of “Cancer Enteral Immunonutrition”

Both malignancies and their treatments very often lead to malnutrition (precisely, sarcopenia and cachexia) and a decline in immune function [39]. Indeed, from 40% to 80% of cancer patients have a state of malnutrition. More in detail, almost 20% of cancer-related deaths are directly attributable to nutritional status derangement [31,32]. Thus, the combined improvement of nutritional status and immune function has become a critical objective considering a comprehensive and multimodal cancer patient care. In this frame, certain targeted nutrients can not only improve nutritional balance but also modulate the immune system machinery [32,35]. These are the background of the growing concept of “cancer immunonutrition” [35]. Subsequently, cancer immunonutrition has gained more efficacy evidence across several clinical settings (e.g., perioperative care, chemo- and radiotherapy, hematopoietic stem cell transplantation (HSCT) setup). To date, enteral immunonutrition has also been used for the management of tumor-related complications [36,37]. In fact, benefits upon enteral immunonutrients’ administration for cancer patients undergoing surgery end up as reduced incidence of infections (local site of infection and systemic ones), improved wound healing, and shorter hospital stay.

Recently, several guidelines on cancer immunonutrition have been published to promote its standardized use in primary and secondary clinical settings [32,37]. The goal of these shared documents is to provide clinicians with a clear and standardized approach for applying immunonutrition in cancer care. This can lead to enhanced patient outcomes and also overcomes immunonutrition application in oncology practice, improving quality of life [40].

### 3.2. Clinical Applications of Enteral Immunonutrition in Gastrointestinal Cancer

#### 3.2.1. Rationale for Perioperative Use of Enteral Immunonutrition in Gastrointestinal Cancer Surgery

Patients with GI tract cancer have a significant association with malnutrition development. The latter can be negatively affected by surgery, chemotherapy, immunotherapy, and more generally, post-treatment complications [36]. Further, malnutrition is a risk factor for immune system depression, derangement of inflammatory response, and the occurrence of surgery complications due to dysregulated stress reaction [37]. Thus, these patients very often have a poor clinical outcome characterized by tumor progression, immune system depressive state, detrimental systemic inflammatory response syndrome (SIRS), pneumonitis occurrence, and wound healing issues [41]. For all these reasons, nutritional support via enteral or parenteral feeding is a standard of care and adjuvant therapy for malnourished patients [42]. Clearly, the selection of parenteral nutrition (PN) or enteral nutrition (EN) depends on the patient’s gastrointestinal function and tolerance of the nutrient formula provided [43]. Regarding the enteral immunonutrition (IN)-based formula, it has been used in patients undergoing elective gastrointestinal cancer surgery. In fact, several randomized controlled trials and meta-analyses support the use of enteral immunonutrition containing arginine, omega-3 fatty acids, glutamine, and nucleotides during the perioperative period [44,45]. For example, a comprehensive meta-analysis of 19 RCTs (2331 patients) found that perioperative enteral IN reduced the occurrence of 56% (RR 0.44) and significantly decreased non-infective complications. Interestingly, hospital stay was shortened by approximately 2.6 days [32,46,47]. Further, EIN was able to maintain effective CD4^+^ T-cell levels with a significant reduction in postoperative SIRS duration [32].

#### 3.2.2. Impact of Enteral Immunonutrition on Immune System Functioning and Postoperative Complications in GI Cancer

Enteral immunonutrition can preserve or amplify key immune indicators following surgery.

We try to distinguish evidence from literature according to the anatomical location of cancer, starting from upper- and following with lower-GI tract cancers.

In detail and in a pooled analysis of upper and lower GI tumor surgeries (namely, out of 4825 patients and 48 RCTs, 19 had upper GI cancer, 9 had lower, and 8 had mixed cancer, whereas 12 had head and neck cancers). Eight studies were conducted preoperatively, 18 were postoperatively, and 22 were in the perioperative period. Precisely, perioperative EIN use comprises the preoperative and postoperative phases of the intervention. EIN reduced the total postoperative complications (relative risk ratio: 0.78; 95% CI, 0.66–0.93; certainty of evidence: high) and infectious complications (surgical site of intervention, systemic infections including pneumonitis and sepsis) (relative risk ratio: 0.71; 95% CI, 0.61–0.82; certainty of evidence: high) vs. standard nutrition [46]. No effects on mortality or adverse events were recorded.

In more detail, specific nutrients showed specific immunomodulatory benefits. Omega-3 fatty acids can up-regulate the number of T-cell subsets (CD3^+^, CD4^+^, and CD4^+^/CD8^+^), the production of immunoglobulins (IgA, IgM, and IgG), and, conversely, reduce pro-inflammatory cytokines (IL-6, TNF-α, and CRP) [33]. Similarly, glutamine and arginine-based formulas can shorten hospital stay and, importantly, reduce infectious complications among colorectal cancer patients [48].

Regarding the quality of the reviewed data, we must observe that for postoperative complications, there is a low ROB for incomplete outcome data and selective reporting, and a low ROB or “some concerns” for random sequence generation and allocation concealment. The ROB for the participant and personnel blinding and outcome assessment are low, “some concerns”, or high, respectively. Quality of meta-analysis indicated minimal publication bias in the reporting of total postoperative complications, infectious and non-infectious complications, mortality rate, length of hospital stay, and side effects/adverse events of EIN.

Gastric cancer is one of the most prevalent malignancies of the GI tract, frequently associated with malnutrition that can be further aggravated by elective surgery [47]. Typically, malnourished gastric cancer patients are at increased risk of adverse postoperative outcomes, including a higher incidence of infectious complications, delayed/impaired wound healing, and prolonged hospital stay [49,50]. In this frame, a comprehensive meta-analysis of seven studies involving 583 patients was conducted (five out of seven trials were done to compare the EIN with standard nutrition, one trial was for comparing EIN with oral placebo, and one trial was for comparing EIN with a regular diet). Most studies included more than one immunonutrient (namely, arginine, glutamine, omega-3 and nucleotides), with the remainder one study conducted with glutamine only. Most studies used the EIN after surgery, and two administered EIN before the operation. Infectious complications such as SIRS (MD, –0.89 days; 95% CI, –1.40 to –0.39; *p* = 0.005) and postoperative complications (RR, 0.29; 95% CI, 0.14–0.60; *p* = 0.001) were significantly reduced in the EIN group. Pulmonary infection and length of hospitalization (LHS) were not significantly improved [51].

Regarding the quality of the meta-analysis, it was figured out the presence of publication bias related to the length of hospital stay (t = −1.98, *p* = 0.186), surgical fistulas occurrence (t = 0.32, *p* = 0.756), wound infections occurrence (t = −0.03, *p* = 0.976) or other infections (t = 0.12, *p* = 0.991). Importantly, neither heterogeneity nor publication bias was found among studies.

Nine publications on the use of enteral and parenteral immunonutrition in colorectal cancer patients were included in a recent meta-analysis. Nine studies provided a total of 1004 patients, including 866 participants receiving the EIN vs. standard enteral formula nutrition support, and 138 received the parenteral immunonutrition formula vs. standard parenteral formula. Immunonutrition use was perioperative in four studies, preoperative in one, and postoperative in four. In the EIN group, the ratio of the control group to the intervention group was 433:433. Considering the six studies on EIN (composed by nucleotides, omega-3 fatty acids, with a total dose ranging from 3 to 15.8 g/day), EIN improved infectious complications (pooled OR, 0.33; 95% CI, 0.21–0.53) which included the surgical site infections (pooled OR, 0.25; 95% CI, 0.22–0.58) and superficial/deep incisional infections ratio (pooled OR, 0.27; 95% CI, 0.12–0.64) and the length of hospital stay (pooled MD, 2.53; 95% CI, 1.29–3.41). In further detail, the EIN group had a shorter length of hospital stay than the standard enteral nutrition formula group: fixed-effect pooled MD was 2.35 (95% CI, 1.29–3.41) with null heterogeneity (*I*^2^ = 0%). Moreover, infectious complications were reduced in the EIN group for the fixed-effect pooled OR 0.33 (95% CI, 0.21–0.53); no heterogeneity was detected (*I*^2^ = 0%). Surgical site infections and superficial/deep incisional infections were reduced in EIN group, with a fixed-effect pooled OR of 0.25 (95% CI, 0.11–0.58) in surgical site infections and of 0.27 (95% CI, 0.12–0.64) in the ration superficial/deep incisional infections; no heterogeneity was detected also (*I*^2^ = 0%) [7,32,48,52].

Regarding the quality of meta-analysis, most studies had a clear description of their random sequence generation (three studies used a computer random number generator, one used an envelope, two used web-based randomization, and three studies did not give sufficient information). Among these, three studies appropriately performed the allocation concealment. Blinding of participants and personnel was conducted in four studies, and blinding of participants and personnel was conducted in five studies. The remaining studies had no sufficient information about blinding. Two studies reported the drop-out before conducting immunonutrition, and the corresponding domain was graded as low risk. All nine included studies showed the pre-specified outcomes in the pre-specified way.

However, the literature evidence appears to be inconsistent for several reasons: different nutrients (and their combinations) and formulas used, together with different regimens (preoperative vs. postoperative)/duration of EIN administration; patients’ populations being heterogeneous, with different complication occurrence/monitoring [8].

Indeed, we must recognize that several meta-analyses bring moderate-quality evidence supporting a promising systematic perioperative use of enteral immunonutrition in GI oncology. Its use shows significant benefits for reducing infection rate, surgical complications, such as anastomotic failures, and shortening recovery time. We must note that the most consistent and strong evidence supporting these summarizing remarks comes from reviewed studies on gastric and colorectal cancer patients.

In addition, formulas containing arginine, omega-3 fatty acids, nucleotides, and glutamine appear more effective in the field of application when used within the ERAS protocol [50]. In fact, standard EN formulas provide essential macronutrients (e.g., protein, fat, and carbohydrates) along with vital micronutrients (e.g., vitamins and minerals). However, clinical outcomes of GI surgery patients under standard EN formulas are not comparable with those under EIN [51,53].

Table 1 reports the main studies reviewed on EIN use in esophageal, gastric and colorectal cancer patients.

#### 3.2.3. Immunonutrition Formulations and Timing of Administration

Converging evidence indicates that the most effective enteral immunonutrition regimens combine arginine, omega-3 fatty acids, and nucleotides when administered in the perioperative period. Interestingly, a meta-analysis on arginine-omega-3 combinations (23 studies (n = 2508; 62% of males)), administered either pre- or postoperatively in 17 of the 23 studies (immunonutrition was administered preoperatively in seven studies and postoperatively in ten studies, respectively), showed a significant reduction in infections’ rate (immunonutrition group vs. standard enteral formula (OR: 0.53; 95% CI: 0.41, 0.68)). In addition, a significant reduction in hospital length of stay (mean difference: −2.08; 95% CI: −2.88, −1.28) of both critically ill and surgical patients was registered [71]. Interestingly, both preoperative immunonutrition administration showed a significant reduction in hospital length of stay (mean difference: −1.84; 95% CI: −3.07, −0.62) and postoperative one (mean difference: −3.08; 95% CI: −3.73, −2.43). The overall mortality rate was not significantly different between the immunonutrition and control group (OR: 1.07; 95% CI: 0.75, 1.53), both preoperatively and postoperatively. Looking at RR values, preoperative (RR, 0.58; 95% CI, 0.43–0.78), postoperative (RR, 0.63; 95% CI, 0.52–0.76), and perioperative EIN methods (RR, 0.46; 95% CI, 0.34–0.62) reduced the incidence of postoperative infectious complications compared with a standard enteral nutrition formula. Moreover, perioperative EIN (RR, 0.65; 95% CI, 0.44–0.95) reduced the incidence of postoperative non-infectious complications, and the postoperative (MD, −2.38; 95% CI, −3.4 to −1.31) and perioperative EIN (MD, −2.64; 95% CI, −3.28 to −1.99) also shortened the length of postoperative hospitalization vs. standard enteral nutrition formula.

In this study, initially, the traditional pair-wise meta-analysis to evaluate the comparative effects of 2 individual treatments that can be directly compared was performed. The estimates of dichotomous and continuous data were expressed as relative risk (RR) and mean difference (MD), respectively. The heterogeneity between studies was tested by using the X^2^ test, and the proportion of the overall variation that is attributable to between-study heterogeneity was also estimated by using the *I*^2^ statistic. Substantial heterogeneity was considered unless the value of the *I^2^* statistic was <50%. Interestingly, the proportion of appropriate descriptions of randomization, allocation concealment, and blinding of the study was 48% (13/27), 37% (10/27), and 44%, respectively. All included trials were rated as low bias risk in incomplete outcome data because the authors stated the drop-out reasons in detail and used the intent-to-treat method to analyze the data. The quality of all eligible studies was graded as low bias risk because expected outcomes of interest were all reported in terms of the selective reporting index. The inconsistency plots suggested that the statistical inconsistency was generally low for weight control, as the corresponding CIs included zero.

In a randomized clinical trial enrolling gastric cancer surgery patient (34 patients with gastric adenocarcinoma or gastric GIST undergoing elective curative surgery), a perioperative formula enriched with arginine, glutamine, and omega-3 (20%, 15 g of arginine, 10%, 7.5 g of glutamine, 20%, 6.96 g of omega-3 in 1200 kcal/1200 mL formula) was administered from 3 days prior to until 14 days after surgery. This was associated with a significant improvement in inflammatory markers and immunological functioning indexes. Indeed, clinical outcomes were similar among EIN and standard enteral nutrition administered patients [72]. In detail, infectious complications (namely, sepsis in one patient and intra-abdominal abscess in another patient in the control group), and non-infectious complications developed in the study group. Non-infectious complications were postoperative bleeding in one patient and delayed gastric emptying in five patients in the control group. On the other hand, regarding surgical complications, wound dehiscence developed in one patient and delayed gastric emptying in one patient in the intervention group. In the control group, one patient developed concomitant sepsis and delayed gastric emptying, and another patient experienced concomitant postoperative bleeding and delayed gastric emptying. Despite a higher number of complications in the control group, no statistically significant difference was observed in infectious (0% vs. 11.8%, *p* = 0.485), non-infectious (11.8% vs. 29.4%, *p* = 0.398), or all complications (11.8% vs. 35.3%, *p* = 0.225) between the two groups. Further, a total of eight patients experienced treatment-related adverse events (three (16.7%) versus five (27.8%) patients in the study versus control group, respectively, *p* = 0.691). These included bloating in one patient in each group, which resulted in the patient’s withdrawal from the study. The remaining adverse events were diarrhea, easily managed by adjustment of the osmolarity of the feeding diet. No death occurred in this study.

In contrast, other studies have shown significant benefits in terms of reduced surgical complications incidence: enhanced wound healing, and fewer overall surgical complications when early postoperative EIN (containing arginine, omega-3, and RNA) was administered to gastric cancer patients. However, these data have been systematically reviewed by the authors who documented the presence of a preoperative regimen of EIN administration (namely, 3–7 days prior to surgery) and a postoperative one (namely, 10 to 21 days after). In the review, the authors also observed the prevalence of meta-analyses vs. original studies in the literature [73].

Thus, globally, meta-analyses consistently support the EIN perioperative approach. The latter includes pre- and postoperative feeding. Following a Bayesian network meta-analysis (an alternative to pool direct and indirect or different indirect evidences simultaneously) of 27 RCTs, pair-wise meta-analyses suggested that preoperative (relative risk [RR], 0.58; 95% confidence interval [CI], 0.43–0.78), postoperative (RR, 0.63; 95% CI, 0.52–0.76), and perioperative EIN methods (RR, 0.46; 95% CI, 0.34–0.62) reduced incidence of postoperative infectious complications compared with standard enteral nutrition. Moreover, perioperative EIN (RR, 0.65; 95% CI, 0.44–0.95) also reduced the incidence of postoperative non-infectious complications. This scheme has been shown to be superior to pre- or postoperative administration alone. In addition, the postoperative (mean difference [MD], −2.38; 95% CI, −3.4 to −1.31) and perioperative EIN administration (MD, −2.64; 95% CI, −3.28 to −1.99) also shortened the length of postoperative hospital stay vs. standard enteral nutrition [74]. In the described study, an initial traditional pair-wise meta-analysis to evaluate the comparative effects of two individual treatments that can be directly compared was performed. The heterogeneity between studies was tested by using an X^2^ test, and the proportion of the overall variation that is attributable to between-study heterogeneity was also estimated by using the *I*^2^ statistic. In detail, substantial heterogeneity was considered unless the value of the *I*^2^ statistic was <50%. In the study, the proportion of appropriate descriptions of randomization, allocation concealment, and blinding is the 48% (13/27), 37% (10/27), and 44%, respectively. All included trials were rated as low bias risk in incomplete outcome data because the authors stated the drop-out reasons in detail and used the intent-to-treat method to analyze the data. The quality of all eligible studies was graded as low bias risk because expected outcomes of interest were all reported in terms of the selective reporting index.

When the oral way of feeding is preserved, several pieces of evidence align with those from EIN administration setups. A comprehensive systematic review of 22 RCTs with 2159 solid cancer surgery patients (gastrointestinal and non-gastrointestinal) showed that oral IN reduced overall infectious complications (RR 0.58, 95 per cent CI 0.48 to 0.70; *I*^2^ = 7 per cent; trial sequential analysis-adjusted 95 per cent CI 0.28 to 1.21) and, in particular, surgical site of infection (RR 0.65, 95%, CI 0.50 to 0.85; *I*^2^ = 0%; trial sequential analysis-adjusted 95% CI 0.21 to 2.04). Thirty-day mortality was not altered by immunonutrition (RR 0.69, 0.33 to 1.40; *I*^2^ = 0%) [75]. This is another gray zone from data analysis that deserves further investigation, perhaps with dedicated long-term multicenter studies. Methodologically, we must remark that the majority of studies used an adequate method of randomization (16 of 22, 73%) and allocation (18 of 22, 82%). However, the remaining did not report the method of randomization and allocation and were considered to have an unclear risk of bias. High risk of performance bias was present in half of the studies (11 of 22, 50%) because they were unblinded. Detection bias was unclear in the majority of studies (15 of 22, 68%) and considered low in the remainder (7 of 22, 32%). Risk of attrition bias was low in 18 studies (82%), high in one study (5%), and unclear in the rest. Risk of reporting bias was unknown in the majority of studies (17 of 22, 77%), low in three studies, and high in two studies. Four trials were at high risk of other biases.

Finally, thirteen studies described adverse effects of, or tolerance to, oral EIN: six were limited to tolerance of tube feeding in the postoperative period. Adverse effects were mostly gastrointestinal (bloating and/or diarrhea). No statistically significant differences were observed, except in one study, which found a higher incidence of postoperative diarrhea in the EIN group vs. the control group [76].

Current guidelines recommend 5–7 days of preoperative EIN administration, with a delivery of 500–1000 mL/day to improve both immune system-related and clinical outcomes [55,61].

The preoperative timing of EIN administration appears particularly advantageous because it reinforces the immune system machinery and prepares the body to mitigate the incoming surgical stress. Moreover, continuing EIN in the postoperative period helps sustain the immune stimulation and tissues’ healing processes [49]. Unfortunately, we cannot retrieve precise and consistent data from the literature on the effective EIN duration of administration in the postoperative period (Table 2).

Dosage of immunonutrients reviewed in the literature gives some quantitative indications for formula selection. The recommended dose of arginine for healthy individuals ranges from 5 to 30 g/day [81]. Indeed, arginine supplementation should remain within the 10–30 g/day range [82]. Higher doses could lead to adverse effects.

The recommended daily intake of omega-3 FAs (fatty acids) to maintain optimal physiological function is 450–500 mg/day [83].

Interestingly, the exact nucleotide content of individual food items has not been officially assessed. However, it is estimated that healthy individuals consume approximately 1–2 g/day from diet [84]. Commercially available enteral formulas contain between 1.2 and 2.8 g/L of nucleotides [66].

Finally, the recommended administration of glutamine via enteral nutrition formula is 0.2–0.3 g/kg/day at the beginning of enteral nutrition. In cases of complicated wound healing, glutamine administration should be extended for 10–15 days [85].

Commonly reported side-effects due to EIN administration are generally mild and gastrointestinal, like nausea, vomiting, diarrhea, bloating, and constipation [86]. Potentially, hyperglycaemia must be prevented, especially in patients with diabetes/critically ill individuals with uncontrolled glucose levels. Some studies have shown that in critically ill, ventilated patients, high-dose immunonutrients (namely, glutamine and other antioxidants) can be associated with increased mortality rates. In fact, high doses of immunonutrients (e.g., selenium, glutamine, or arginine) can have systemic toxicity. High doses of omega-3 can affect blood clotting times [68].

In summary, we can consider converging data on EIN perioperative use and formula composition, although with heterogeneity of immunonutrient combinations and amounts among studies. Preoperative EIN administration duration recognizes a more defined time window vs. the postoperative one. GI side-effects and adverse events are the most common in surgical GI patients under EIN. Attention must be paid to the immunonutrient dosage.

## 4. Conclusions

Enteral immunonutrition has gained increasing attention within the scientific community as a supportive strategy, especially in the postoperative management of gastrointestinal cancer patients. Delivering targeted nutrients with immunomodulatory properties (mainly, arginine, omega-3 fatty acids, glutamine, and nucleotides), EIN aims to improve surgical recovery by enhancing host immune function, reducing systemic inflammation, and promoting mucosal integrity. Finally, EIN also covers the enhanced nutritional requirements of cancer and post-surgical patients. Several clinical trials and meta-analyses have reported promising and often but not completely consistent results in patients undergoing head and neck, esophageal, gastric and colonic tract surgery for cancer. In detail, pre-/postoperative and the combined EIN administration have been associated with fewer infectious and non-infectious complications, related improved immunological parameters, and shorter hospital stay. No clear effects on mortality have been described. Regarding the cited meta-analyses, we must underline that they have a good accuracy and low bias risk.

However, we must recognize that EIN’s proven efficacy across all GI tracts undergoing surgery also remains a topic of investigation. In fact, we do not have so many studies in esophageal cancer patients. Moreover, in esophagectomy patients, some evidence has shown comparable outcomes between standard enteral nutrition and EIN, raising questions over patient subgroup stratification and the type of surgery. Moreover, and very interestingly, EIN efficacy in patients with advanced malnutrition (namely, cachexia) or deep immunosuppression is still not clearly defined.

Tailored approaches are warranted and required in order to solve and overcome the issues of heterogeneity of the reviewed evidence (mainly meta-analyses) (namely, due to tumor sites, EIN formulations, timing and duration of administration, comparators, and different perioperative protocols). Despite these spots of gray zone, enteral IN remains a well-tolerated and physiologically aligned nutritional intervention with a favorable safety profile. Mild and mainly gastrointestinal side-effects have been described in the literature.

Future research should aim to clarify optimal nutrient combinations (also with established quantitative cut-off), timing and scheme of administration (pre-, postoperative or both), and duration of treatment. These issues can be addressed via multicentric trials and through matching tumor type, nutritional status, and individual immune system profiling. Perhaps, these aims can also be accomplished with artificial intelligence (AI)-assisted systems. Furthermore, integrating EIN with personalized perioperative care protocols (e.g., ERAS) can lead to improved clinical relevance.

## Figures and Tables

**Table 1:** Main studies reviewed on EIN use in esophageal, gastric and colorectal cancer patients undergoing surgery.

Study Type	Enrolled Population	Clinical Findings	References
RCT	In total, 30 esophageal cancer patients receiving preoperative immunomodulating diet (oral nutrient supplement) (n = 15) vs. standard enteral nutrition (n = 15)	Lower infectious complication rate (pneumonia rate); improved inflammatory markers.	[54]
RCT	In total, 26 esophageal cancer patients receiving preoperative antioxidant-enriched immuno-enhanced diet (n = 14) vs. control diet (n = 12)	Lower infectious complication rate.	[48]
RCT	In total, 300 esophago-gastric patients receiving either preoperative EIN (n = 149) vs. standard formula (n = 151)	No impact on incidence of infectious and non-infectious complications, surgical complications at 30 days, length of hospital stay, or readmission rate.	[45]
RCT	In total, 112 patients with Gastric cancer and cachexia receiving either preoperative EIN (n = 56) or standard enteral nutrition (n = 56)	Significantly lower incidence of postoperative and overall infectious complications; less weight loss, shorter duration of antibiotic use, hospital stay, and total hospital costs vs. standard nutrition.	[55]
Retrospective cohort study	In total, 134 patients Group A: (n = 79) under standard enteral nutrition vs. Group B (n = 55) EIN	In total, 34% reduction in length of hospital stay, and 21% reduction for need of parenteral nutrition; 70.1% reduction for risk of infectious complications; reduced weight loss, need for blood transfusions, and surgical re-intervention.	[56]
RCT	In total, 98 patients under EIN group (n = 44) vs. standard enteral nutrition (n = 54)	Reduced incidence of pulmonary complications in the EIN group; Sixty-day mortality: lower in the EIN group but similar (EIN vs. standard enteral nutrition) 6th and 12th-month survival.	[57]
Randomized phase III clinical trial	In total, 124 patients: Safety: In total, 61 under standard enteral nutrition vs. 63 under EIN (Eicosapentaenoic acid-rich nutrition) Efficacy: In total, 60 under standard enteral nutrition vs. 63 under EIN. Seven days before and 21 days after surgery	Similar surgical morbidity rate (13% vs. 14%); No effect on median bodyweight loss at one month and three months after gastrectomy	[58]
RCT	In total, 99 patients: In total, 54 under standard enteral nutrition vs. 45 under EIN	No impact on overall survival. However, at three months, Nine deaths for standard enteral nutrition vs. no deaths for EIN group. At univariate analyses, EIN group had lower risk of mortality during first year of follow-up. However, EIN did not affect mortality risk when patients were analyzed together.	[59]
RCT	In total, 109 patients under Early postoperative EIN (54 pts) vs. isocaloric–isonitrogenous diet (55 pts)	Lower incidence of postoperative infectious complications in the EIN vs. control group; Lower anastomotic leak rate in the EIN vs. control group; No difference in the mortality rate; Reduced length of hospitalization in EIN vs. control group.	[60]
RCT	In total, 244 patients: EIN group (n = 127) vs. control group (n = 117).	No significant differences for surgical-site infections, overall infectious complications, and overall postoperative morbidity rate.	[61]
RCT	EIN group (n = 30) vs. control group (n = 30) (standard formula) administered preoperatively for seven days	Significantly lower postoperative infectious complications rate in the EIN group.	[62]
RCT	Early postoperative EIN (n = 30) vs. isocaloric–isonitrogenous control nutrition (n = 30)	Lower incidence rate of surgical wound healing complications after EIN vs. the control group.	[6]
RCT	In total, 23 CRC administered with perioperative EIN (n = 12) vs. standard oral formula	No significant differences between groups for infectious, non-infectious complications; no difference for length of hospital stay.	[63]
RCT	In total, 100 CRC patients receiving perioperative EIN (n = 50) vs. standard oral formula (n = 50)	Reduced incidence of infectious complications; reduced length of hospital stay after EIN vs. control.	[64]
Perspective trial	In total, 28 CRC patients receiving perioperative EIN (n = 14) vs. standard formula (n = 14)	No difference in incidence of infectious complications, length of hospital stays after EIN vs. control; sustained rise in CD4 T-cells during the postoperative period after EIN vs. control formula.	[65]
RCT	In total, 161 CRC receiving preoperative EIN (n = 79) vs. standard formula (n = 82)	No difference in incidence of infectious, non-infectious complications, length of hospital stays after EIN vs. control	[66]
RCT	In total, 84 CRC receiving preoperative EIN (n = 42) vs. standard oral formula (n = 42)	In rectal-cancer-only individuals, EIN group had significantly fewer infectious complications vs. control group.	[67]
Perspective trial	In total, 84 CRC receiving preoperative EIN (n = 42) vs. standard oral formula (n = 42)	EIN group had significantly fewer infectious complications vs. control group.	[68]
Perspective trial	In total, 122 CRC receiving perioperative EIN (n = 61) vs. standard oral formula (n = 61)	EIN group had a significant decrease in wound infection rates.	[69]
RCT	In total, 128 CRC receiving perioperative EIN (n = 64) vs. standard oral formula (n = 64)	No difference in incidence of infectious, non-infectious complications, length of hospital stays after EIN vs. control	[70]

Legend: EIN, enteral immunonutrition; RCT, randomized clinical trial; CRC, colorectal cancer patients.

**Table 2:** Main immunonutrients used in enteral immunonutrition and focus on their functions, mechanism of action, and clinical implications in GI cancer patients undergoing surgery.

Nutrient	Primary Function	Mechanism of Action	Clinical Benefits in GI Cancer Surgery	References
Arginine (10–30 g/day range) (oral or EIN, ranging 5–7 days prior to surgery, ranging 10–21 days after)	Enhancing immune system functioning and improving wound healing	Precursor to the synthesis of nitric oxide; support of lymphocyte functioning and of collagen synthesis	↓ Surgical-site infections; ↑ wound healing; ↓ length of stay; moderate level of evidence	[57,59]
Glutamine (0.2–0.3 g/kg/day) (oral or EIN, ranging 5–7 days prior to surgery, ranging 10–21 days after)	Maintenance of gut integrity and supporting of immune cells functioning	Feed for enterocytes and immune cells; reduction in oxidative stress; ↓ mucosal damage; ↑ lymphocyte count	↓ Hospitalization complications (both infectious and non-infectious ones); moderate level of evidence	[58,68]
Omega-3 fatty acids (450–500 mg/day) (oral or EIN, ranging 5–7 days prior to surgery, ranging 10–21 days after)	Combined anti-inflammatory and immune-modulating effects	Modulation of eicosanoid pathway; suppression of pro-inflammatory cytokines synthesis (↓ CRP, IL−6); ↑ T-cell activity	↓ Infectious complications; moderate level of evidence	[58,77]
Nucleotides (1.2–2.8 g/L) (oral or EIN, ranging 5–7 days prior to surgery, ranging 10–21 days after)	Support of immune cells’ proliferation and tissue repair	Enhanced DNA/RNA synthesis during cellular replication/repairing (↑ lymphocyte count)	Enhanced recovery in the postsurgery period; ↑ healing rate; moderate level of evidence	[58,78,79]
Selenium, vitamins (A, C, E) (200 microg selenium, 80 mg vitamin C, 24 mg vitamin E, and 400–800 microg vitamin A) (oral or EIN, ranging 5–7 days prior to surgery)	Antioxidant effect and co-factors providing for effective immune responses	Scavengers to free radicals (↓ oxidative stress); modulation of inflammation/leukocyte activation (↑ immunoglobulin production)	↓ Inflammatory complications; low level of evidence	[58,80]

Table legend: ↓: decrease and ↑: increase.

## Data Availability

All the data reviewed in the manuscript can be retrieved from the main medical database (e.g., PubMed, Medline) and from the websites of the most important gastroenterology and nutrition international meetings (e.g., UEGW, DDW, SINPE, and ESPEN).

## References

[1] Donlon N.E., Davern M., Hayes C., Power R., Sheppard A.D., Donohoe C.L., Lysaght J., Reynolds J.V. (2022). The immune response to major gastrointestinal cancer surgery and potential implications for adjuvant immunotherapy. Crit. Rev. Oncol. Hematol..

[2] Xin C., Wang Y., Luo Y., Gai Y., Han B. (2025). Effect of perioperative immunonutrition on outcomes in gastric cancer surgery patients: A systematic review and evidence map. Clin. Nutr. ESPEN.

[3] Chen C., Hu X., He D., He X., Shen L. (2025). Nutritional Immunity in Wound Infection: Unveiling the Role of Dietary Elements in Host-Pathogen Interaction. Food Sci. Nutr..

[4] Cheng M., Li Y., Liang D., Wu C. (2025). Synergistic power of functional foods and exercise in colorectal cancer control: Targeting metabolism, mitochondrial function, redox homeostasis, exercise performance, neuroimmune signaling, and brain-gut axis crosstalk. Front. Nutr..

[5] Martínez-Augustin O., Tena-Garitaonaindia M., Ceacero-Heras D., Jiménez-Ortas Á., Enguix-Huete J.J., Álvarez-Mercado A.I., Ruiz-Henares G., Aranda C.J., Gámez-Belmonte R., Sánchez de Medina F. (2025). Macronutrients as Regulators of Intestinal Epithelial Permeability: Where Do We Stand?. Compr. Rev. Food Sci. Food Saf..

[6] Farreras N., Artigas V., Cardona D., Rius X., Trias M., González J.A. (2005). Effect of early postoperative enteral immunonutrition on wound healing in patients undergoing surgery for gastric cancer. Clin. Nutr..

[7] Wong C.S., Aly E.H. (2016). The effects of enteral immunonutrition in upper gastrointestinal surgery: A systematic review and meta-analysis. Int. J. Surg..

[8] Khan A., Wong J., Riedel B., Laing E., Beaumont A., Kong J., Warrier S., Heriot A. (2023). The Impact of Peri-Operative Enteral Im-munonutrition on Post-Operative Complications in Gastrointestinal Cancer Surgery: A Meta-Analysis. Ann. Surg. Oncol..

[9] Senkal M., Zumtobel V., Bauer K.H., Marpe B., Wolfram G., Frei A., Eickhoff U., Kemen M. (1999). Outcome and cost-effectiveness of perio erative enteral immunonutrition in patients undergoing elective upper gastrointestinal tract surgery: A prospective randomized study. Arch. Surg..

[10] Buddrus U., Kutza J.O., Thye J., Esdar M., Hübner U.H., Liebe J.D. (2026). PICO-based assessment and categorization of evidence for digital health interventions: An inductive framework development. Front. Digit. Health.

[11] Dindo D., Demartines N., Clavien P.A. (2004). Classification of surgical complications: A new proposal with evaluation in a cohort of 6336 patients and results of a survey. Ann. Surg..

[12] Menezes T.M., Dias M.O., Dos Reis R., Elias J., Lucchesi F.R., Araujo R.L.C. (2020). Prognostic value of muscle depletion assessed by computed tomography for surgical outcomes of cancer patients undergoing total esophagectomy and gastrectomy. J. Surg. Oncol..

[13] Page M.J., Moher D., Bossuyt P.M., Boutron I., Hoffmann T.C., Mulrow C.D., Shamseer L., Tetzlaff J.M., Akl E.A., Brennan S.E. (2021). PRISMA 2020 explanation and elaboration: Updated guidance and exemplars for reporting systematic reviews. BMJ.

[14] Soeters P.B., Grimble R.F. (2009). Dangers, and benefits of the cytokine mediated response to injury and infection. Clin. Nutr..

[15] Koo T.H., Leong X.B., Zakaria A.D. (2024). Current Trend and Outcomes on Immunonutrition in Medical and Surgical Fields: An Updated Perspective. Malays. J. Med. Sci..

[16] Philpott M., Ferguson L.R. (2004). Immunonutrition and cancer. Mutat. Res..

[17] Yuen R.C., Tsao S.Y. (2021). Embracing cancer immunotherapy with vital micronutrients. World J. Clin. Oncol..

[18] Alexander J.W. (1993). Immunoenhancement via enteral nutrition. Arch. Surg..

[19] McCowen K.C., Bistrian B.R. (2003). Immunonutrition: Problematic or problem solving?. Am. J. Clin. Nutr..

[20] Agarwal R. (2000). Cell signaling and regulators of cell cycle as molecular targets for prostate cancer prevention by dietary agents. Bio-chem. Pharmacol..

[21] Ham H.J., Kim J. (2025). Targeted nutritional strategies in postoperative care. Anesth. Pain Med..

[22] Bang H.O., Dyerberg J., Sinclair H.M. (1980). The composition of the Eskimo food in north western Greenland. Am. J. Clin. Nutr..

[23] Puertollano M.A., Puertollano E., Alvarez de Cienfuegos G., de Pablo M.A. (2007). Significance of olive oil in the host immune resistance to infection. Br. J. Nutr..

[24] Ferreira C., Vieira P., Sá H., Malva J., Castelo-Branco M., Reis F., Viana S. (2024). Polyphenols: Immunonutrients tipping the balance of immunometabolism in chronic diseases. Front. Immunol..

[25] Yu X., Yu H., Yang C., Wu C., Cui Y., Xu N. (2025). Gellan gum/chitosan-based bilayer scaffold for the targeted delivery of curcumin and green tea, designed to enhance breast cancer treatment paradigms. Carbohydr. Polym..

[26] Kamocki Z., Matowicka-Karna J., Jurczuk A., Milewska A., Niewinski A., Zareba K., Kedra B. (2023). Preoperative Glutamine Supplementation in Gastric Cancer—Thrombocyte Phagocytic Activity and Early Postoperative Outcomes. Nutrients.

[27] Maulydia M., Rehatta N.M., Soedarmo S.M. (2023). Effects of glutamine and arginine combination on pro- and anti-inflammatory cytokines. Open Vet. J..

[28] Kavalukas S., McClave S.A. (2023). Immunonutrition vs standard nutrition for patients with cancer. Nutr. Clin. Pract..

[29] Grimble R.F. (2005). Immunonutrition. Curr. Opin. Gastroenterol..

[30] Blum S., Haller D., Pfeifer A., Schiffrin E.J. (2002). Probiotics and immune response. Clin. Rev. Allergy Immunol..

[31] Liu X., Chen Y., Zhang S., Dong L. (2021). Gut microbiota-mediated immunomodulation in tumor. J. Exp. Clin. Cancer Res..

[32] Khajoueinejad N., Santiago C., Turner K., Pimiento J.M. (2025). Nutritional Support for Gastrointestinal Cancer Patients: New (and Old) Frontiers in Management, a Narrative Review. Nutrients.

[33] Ye J., Hu Y., Chen X., Chang C., Li K. (2023). Comparative Effects of Different Nutritional Supplements on Inflammation, Nutritional Status, and Clinical Outcomes in Colorectal Cancer Patients: A Systematic Review and Network Meta-Analysis. Nutrients.

[34] Ma M., Zheng Z., Zeng Z., Li J., Ye X., Kang W. (2023). Perioperative Enteral Immunonutrition Support for the Immune Function and Intestinal Mucosal Barrier in Gastric Cancer Patients Undergoing Gastrectomy: A Prospective Randomized Controlled Study. Nutrients.

[35] Yu K., Zheng X., Wang G., Liu M., Li Y., Yu P., Yang M., Guo N., Ma X., Bu Y. (2020). Immunonutrition vs Standard Nutrition for Cancer Patients: A Systematic Review and Meta-Analysis (Part 1). JPEN J. Parenter. Enter. Nutr..

[36] Gyan E., Raynard B., Durand J.P., Lacau Saint Guily J., Gouy S., Movschin M.L., Khemissa F., Flori N., Oziel-Taieb S., Bannier Brati-cevic C. (2018). Malnutrition in Patients with Cancer: Comparison of Perceptions by Patients, Relatives, and Physicians-Results of the NutriCancer2012 Study. JPEN J. Parenter. Enter. Nutr..

[37] Klek S. (2011). Immunonutrition in cancer patients. Nutrition.

[38] Hussein M., Mohamed T., Mubarak F.A., Morad F., Naser M., Humaira A., Amina H., Khan M. (2025). Nutritional support and immunonutrition in esophageal cancer—From perioperative care to long-term survivorship: A review. Biomol. Biomed..

[39] Singh S.K., Dorak M.T. (2017). Cancer Immunoprevention and Public Health. Front. Public Health.

[40] Ruggeri E., Ostan R., Varani S., Pannuti R., Biasco G. (2022). Home Artificial Nutrition and Energy Balance in Cancer Patients: Nutri-tional and Clinical Outcomes. Nutrients.

[41] Zhang Y., Gu Y., Guo T., Li Y., Cai H. (2012). Perioperative immunonutrition for gastrointestinal cancer: A systematic review of randomized controlled trials. Surg. Oncol..

[42] Oodit R., Biccard B.M., Panieri E., Alvarez A.O., Sioson M.R.S., Maswime S., Thomas V., Kluyts H.L., Peden C.J., de Boer H.D. (2022). Guidelines for Perioperative Care in Elective Abdominal and Pelvic Surgery at Primary and Secondary Hospitals in Low-Middle-Income Countries (LMIC’s): Enhanced Recovery After Surgery (ERAS) Society Recommendation. World J. Surg..

[43] Herbert G., Perry R., Andersen H.K., Atkinson C., Penfold C., Lewis S.J., Ness A.R., Thomas S. (2019). Early enteral nutrition within 24 hours of lower gastrointestinal surgery versus later commencement for length of hospital stay and postoperative complications. Cochrane Database Syst. Rev..

[44] Markar S., Mariette C., Bonnetain F., Lundell L., Rosati R., de Manzoni G., Bonavina L., Tucker O., Plum P., D’Journo X.B. (2025). Immunonutrition to improve the quality of life of upper gastrointestinal cancer patients undergoing neoadjuvant treatment prior to surgery (NEOIMMUNE): Double-blind randomized con-trolled multicenter clinical trial. Dis. Esophagus.

[45] Kaniel O., Sherf-Dagan S., Szold A., Langer P., Khalfin B., Kessler Y., Raziel A., Sakran N., Motro Y., Goitein D. (2022). The Effects of One Anastomosis Gastric Bypass Surgery on the Gastrointestinal Tract. Nutrients.

[46] Maurício S.F., Xiao J., Prado C.M., Gonzalez M.C., Correia M.I.T.D. (2018). Different nutritional assessment tools as predictors of postoperative complications in patients undergoing colorectal cancer resection. Clin. Nutr..

[47] Klek S., Laviano A., Chrostek H., Cardenas D. (2025). Nutrition in Oncology: Overcoming Challenges to Optimize the Patient Journey from Prehabilitation to Rehabilitation. Oncol. Ther..

[48] Xin F., Mzee S.A.S., Botwe G., He H., Zhiyu S., Gong C., Said S.T., Jixing C. (2019). Short-term evaluation of immune levels and nutritional values of EN versus PN in gastric cancer: A systematic review and a meta-analysis. World J. Surg. Oncol..

[49] Aprile G., Basile D., Giaretta R., Schiavo G., La Verde N., Corradi E., Monge T., Agustoni F., Stragliotto S. (2021). The Clinical Value of Nutritional Care before and during Active Cancer Treatment. Nutrients.

[50] Vidal-Casariego A., Calleja-Fernández A., Villar-Taibo R., Kyriakos G., Ballesteros-Pomar M.D. (2014). Efficacy of arginine-enriched enteral formulas in the reduction of surgical complications in head and neck cancer: A systematic review and meta-analysis. Clin. Nutr..

[51] McKay B.P., Larder A.L., Lam V. (2019). Pre-Operative vs. Peri-Operative Nutrition Supplementation in Hepatic Resection for Cancer: A Systematic Review. Nutr. Cancer.

[52] Bossi P., De Luca R., Ciani O., D’Angelo E., Caccialanza R. (2022). Malnutrition management in oncology: An expert view on controversial issues and future perspectives. Front. Oncol..

[53] Matsui R., Sagawa M., Sano A., Sakai M., Hiraoka S.I., Tabei I., Imai T., Matsumoto H., Onogawa S., Sonoi N. (2024). Impact of Perioperative Immunonutrition on Postoperative Outcomes for Patients Undergoing Head and Neck or Gastrointestinal Cancer Surgeries: A Systematic Review and Meta-Analysis of Randomized Controlled Trials. Ann. Surg..

[54] Cheng Y., Zhang J., Zhang L., Wu J., Zhan Z. (2018). Enteral immunonutrition versus enteral nutrition for gastric cancer patients under-going a total gastrectomy: A systematic review and meta-analysis. BMC Gastroenterol..

[55] Xu J., Sun X., Xin Q., Cheng Y., Zhan Z., Zhang J., Wu J. (2018). Effect of immunonutrition on colorectal cancer patients undergoing surgery: A meta-analysis. Int. J. Color. Dis..

[56] De Luca R., Gianotti L., Pedrazzoli P., Brunetti O., Rizzo A., Sandini M., Paiella S., Pecorelli N., Pugliese L., Pietrabissa A. (2023). Immunonutrition and prehabilitation in pancreatic cancer surgery: A new concept in the era of ERAS^®^ and neoadjuvant treatment. Eur. J. Surg. Oncol..

[57] Weimann A., Braga M., Harsanyi L., Laviano A., Ljungqvist O., Soeters P., Jauch K.W., Kemen M., Hiesmayr J.M., DGEM (German Society for Nutritional Medicine) (2006). ESPEN Guidelines on Enteral Nutrition: Surgery including organ transplantation. Clin. Nutr..

[58] Heyland D.K., Novak F., Drover J.W., Jain M., Su X., Suchner U. (2001). Should immunonutrition become routine in critically ill patients? A systematic review of the evidence. JAMA.

[59] Kitagawa H., Namikawa T., Yatabe T., Munekage M., Yamasaki F., Kobayashi M., Hanazaki K. (2017). Effects of a preoperative immune-modulating diet in patients with esophageal cancer: A prospective parallel group randomized study. Langenbecks Arch. Surg..

[60] Aiko S., Kumano I., Yamanaka N., Tsujimoto H., Takahata R., Maehara T. (2012). Effects of an immuno-enhanced diet containing antioxidants in esophageal cancer surgery following neoadjuvant therapy. Dis. Esophagus.

[61] Yu J., Yuan A., Liu Q., Wang W., Sun Y., Li Z., Meng C., Zhou Y., Cao S. (2024). Effect of preoperative immunonutrition on post-operative short-term clinical outcomes in patients with gastric cancer cachexia: A prospective randomized controlled trial. World J. Surg. Oncol..

[62] Martínez González Á., Llópiz Castedo J., Rodeiro Escobar P., González Nunes M., Fernández López B., García Cardoner M.L.Á., Fraile Amador F.J., Rodriguez Zorrilla S., Martínez González M.I., Rodeiro Marta S.E. (2024). Effectiveness of immunonutrition-in the perioperative nutritional management of gastric cancer. Nutr. Hosp..

[63] Scislo L., Pach R., Nowak A., Walewska E., Gadek M., Brandt P., Puto G., Szczepanik A.M., Kulig J. (2018). The impact of post-operative enteral immunonutrition on postoperative complications and survival in gastric cancer patients—Randomized clinical trial. Nutr. Cancer.

[64] Ida S., Hiki N., Cho H., Sakamaki K., Ito S., Fujitani K., Takiguchi N., Kawashima Y., Nishikawa K., Sasako M. (2017). Randomized clinical trial comparing standard diet with perioperative oral immunonutrition in total gastrectomy for gastric cancer. Br. J. Surg..

[65] Klek S., Scislo L., Walewska E., Choruz R., Galas A. (2017). Enriched enteral nutrition may improve short-term survival in stage IV gastric cancer patients: A randomized, controlled trial. Nutrition.

[66] Marano L., Porfidia R., Pezzella M., Grassia M., Petrillo M., Esposito G., Braccio B., Gallo P., Boccardi V., Cosenza A. (2013). Clinical and immunological impact of early postoperative enteral immunonutrition after total gastrectomy in gastric cancer patients: A prospective randomized study. Ann. Surg. Oncol..

[67] Fujitani K., Tsujinaka T., Fujita J., Miyashiro I., Imamura H., Kimura Y., Kobayashi K., Kurokawa Y., Shimokawa T., Furukawa H. (2012). Prospective randomized trial of preoperative enteral immunonutrition followed by elective total gastrectomy for gastric cancer. Br. J. Surg..

[68] Wilhelm S.M., Kale-Pradhan P.B. (2010). Combination of arginine and omega-3 fatty acids enteral nutrition in critically ill and surgical patients: A meta-analysis. Expert Rev. Clin. Pharmacol..

[69] Okamoto Y., Okano K., Izuishi K., Usuki H., Wakabayashi H., Suzuki Y. (2009). Attenuation of the systemic inflammatory response and infectious complications after gastrectomy with preoperative oral arginine and omega-3 fatty acids supplemented immunonutrition. World J. Surg..

[70] Angka L., Martel A.B., Ng J., Pecarskie A., Sadiq M., Jeong A., Scaffidi M., Tanese de Souza C., Kennedy M.A., Tadros S. (2022). A Translational Randomized Trial of Perioperative Arginine Immunonutrition on Natural Killer Cell Function in Colorectal Cancer Surgery Patients. Ann. Surg. Oncol..

[71] Braga M., Gianotti L., Vignali A., Carlo V.D. (2002). Preoperative oral arginine and n-3 fatty acid supplementation improves the im-munometabolic host response and outcome after colorectal resection for cancer. Surgery.

[72] Finco C., Magnanini P., Sarzo G., Vecchiato M., Luongo B., Savastano S., Bortoliero M., Barison P., Merigliano S. (2007). Prospective randomized study on perioperative enteral immunonutrition in laparoscopic colorectal surgery. Surg. Endosc..

[73] Lee S.Y., Lee J., Park H.M., Kim C.H., Kim H.R. (2023). Impact of Preoperative Immunonutrition on the Outcomes of Colon Cancer Surgery: Results from a Randomized Controlled Trial. Ann. Surg..

[74] Manzanares Campillo M.D.C., Martín Fernández J., Amo Salas M., Casanova Rituerto D. (2017). A randomized controlled trial of pre-operative oral immunonutrition in patients undergoing surgery for colorectal cancer: Hospital stay and health care costs. Cir. Cir..

[75] Moya P., Miranda E., Soriano-Irigaray L., Arroyo A., Aguilar M.D., Bellón M., Muñoz J.L., Candela F., Calpena R. (2016). Perioperative im-munonutrition in normo-nourished patients undergoing laparoscopic colorectal resection. Surg. Endosc..

[76] Sorensen L.S., Thorlacius-Ussing O., Schmidt E.B., Rasmussen H.H., Lundbye-Christensen S., Calder P.C., Lindorff-Larsen K. (2014). Randomized clinical trial of perioperative omega-3 fatty acid supplements in elective colorectal cancer surgery. Br. J. Surg..

[77] Ma C., Tsai H., Su W., Sun L., Shih Y., Wang J. (2018). Combination of arginine, glutamine, and omega-3 fatty acid supplements for peri-operative enteral nutrition in surgical patients with gastric adenocarcinoma or gastrointestinal stromal tumor (GIST): A prospective, randomized, double-blind study. J. Postgrad. Med..

[78] Triantafillidis J.K., Malgarinos K. (2024). Immunonutrition in Operated-on Gastric Cancer Patients: An Update. Biomedicines.

[79] Song G.M., Tian X., Zhang L., Ou Y.X., Yi L.J., Shuai T., Zhou J.G., Zeng Z., Yang H.L. (2015). Immunonutrition Support for Patients Undergoing Surgery for Gastrointestinal Malignancy: Preoperative, Postoperative, or Perioperative? A Bayesian Network Meta-Analysis of Randomized Controlled Trials. Medicine.

[80] Buzquurz F., Bojesen R.D., Grube C., Madsen M.T., Gögenur I. (2020). Impact of oral preoperative and perioperative immunonutrition on postoperative infection and mortality in patients undergoing cancer surgery: Systematic review and meta-analysis with trial sequential analysis. BJS Open..

[81] Hamilton-Reeves J.M., Bechtel M.D., Hand L.K., Schleper A., Yankee T.M., Chalise P., Lee E.K., Mirza M., Wyre H., Griffin J. (2016). Effects of Immunonutrition for Cystectomy on Immune Response and Infection Rates: A Pilot Randomized Controlled Clinical Trial. Eur. Urol..

[82] Matsui R., Sagawa M., Inaki N., Fukunaga T., Nunobe S. (2024). Impact of Perioperative Immunonutrition on Postoperative Outcomes in Patients with Upper Gastrointestinal Cancer: A Systematic Review and Meta-Analysis of Randomized Controlled Trials. Nutrients.

[83] Goyal A., Macias C.A., Corzo M.P., Vargas V.P.S., Mendoza M., Guarecuco Castillo J.E., Garcia A., Morfin-Meza K.D., Fuentes-Orozco C., González-Ojeda A. (2025). Perioperative Immunonutrition in Gastrointestinal Oncology: A Comprehensive Umbrella Review and Meta-Analysis on Behalf of TROGSS-The Robotic Global Surgical Society. Nutrients.

[84] Mauskopf J.A., Candrilli S.D., Chevrou-Séverac H., Ochoa J.B. (2012). Immunonutrition for patients undergoing elective surgery for gastrointestinal cancer: Impact on hospital costs. World J. Surg. Oncol..

[85] Santos S.S., da Costa L.A.T.J., Araripe T.S.O., Reges B.D.L.O., Ximenes H.M.A., Moreira A.C.O.M. (2025). Plasma proteomic profiles reveal immune modulation by immunonutrition in GI cancer. Nutrition.

[86] Sodergren M.H., Jethwa P., Kumar S., Duncan H.D., Johns T., Pearce C.B. (2010). Immunonutrition in patients undergoing major upper gastrointestinal surgery: A prospective double-blind randomised controlled study. Scand. J. Surg..

